# A prospective randomized controlled multicenter trial comparing antibiotic therapy with appendectomy in the treatment of uncomplicated acute appendicitis (APPAC trial)

**DOI:** 10.1186/1471-2482-13-3

**Published:** 2013-02-08

**Authors:** Hannu Paajanen, Juha M Grönroos, Tero Rautio, Pia Nordström, Markku Aarnio, Tuomo Rantanen, Saija Hurme, Kirsti Dean, Airi Jartti, Jukka-Pekka Mecklin, Juhani Sand, Paulina Salminen

**Affiliations:** 1 Department of Surgery, Kuopio University Hospital, Kuopio, Finland; 2 Department of Surgery, Mikkeli Central Hospital and Institute of clinical medicine, Mikkeli, Finland; 3 University of Eastern Finland, Kuopio, Finland; 4 Department of Surgery, Turku University Hospital, Turku, Finland; 5 University of Turku, Turku, Finland; 6 Department of Surgery, Oulu University Hospital, Oulu, Finland; 7 Division of Surgery, Gastroenterology and Oncology, Tampere University Hospital, Tampere, Finland; 8 Department of Surgery, Jyväskylä Central Hospital, Jyväskylä, Finland; 9 Department of Surgery, Seinäjoki Central Hospital, Seinäjoki, Finland; 10 Department of Biostatistics, University of Turku, Turku, Finland; 11 Department of Radiology, Turku University Hospital, Turku, Finland; 12 Department of Radiology, Oulu University Hospital, Oulu, Finland

**Keywords:** Acute appendicitis, Appendicitis, Uncomplicated appendicitis, Appendectomy, Appendicectomy, Antibiotic treatment, Conservative, Non-operative, Randomized

## Abstract

**Background:**

Although the standard treatment of acute appendicitis (AA) consists of an early appendectomy, there has recently been both an interest and an increase in the use of antibiotic therapy as the primary treatment for uncomplicated AA. However, the use of antibiotic therapy in the treatment of uncomplicated AA is still controversial.

**Methods/design:**

The APPAC trial is a randomized prospective controlled, open label, non-inferiority multicenter trial designed to compare antibiotic therapy (ertapenem) with emergency appendectomy in the treatment of uncomplicated AA. The primary endpoint of the study is the success of the randomized treatment. In the antibiotic treatment arm successful treatment is defined as being discharged from the hospital without the need for surgical intervention and no recurrent appendicitis during a minimum follow-up of one-year (treatment efficacy). Treatment efficacy in the operative treatment arm is defined as successful appendectomy evaluated to be 100%. Secondary endpoints are post-intervention complications, overall morbidity and mortality, the length of hospital stay and sick leave, treatment costs and pain scores (VAS, visual analoque scale). A maximum of 610 adult patients (aged 18–60 years) with a CT scan confirmed uncomplicated AA will be enrolled from six hospitals and randomized by a closed envelope method in a 1:1 ratio either to undergo emergency appendectomy or to receive ertapenem (1 g per day) for three days continued by oral levofloxacin (500 mg per day) plus metronidazole (1.5 g per day) for seven days. Follow-up by a telephone interview will be at 1 week, 2 months and 1, 3, 5 and 10 years; the primary and secondary endpoints of the trial will be evaluated at each time point.

**Discussion:**

The APPAC trial aims to provide level I evidence to support the hypothesis that approximately 75–85% of patients with uncomplicated AA can be treated with effective antibiotic therapy avoiding unnecessary appendectomies and the related operative morbidity, also resulting in major cost savings.

**Trial registration:**

Clinicaltrials.gov http://NCT01022567

## Background

Emergency appendectomy for acute appendicitis (AA) is an effective and universally accepted procedure performed more than 300,000 times annually in the United States [1]. The life-time risk to have AA is 8.6% in men and 6.7% in women; the risk for emergency appendectomy is 12% and 23%, respectively [2]. In Finland, approximately 6,500 appendectomies are performed annually with a mean hospital stay of 2.7 days [3]. For over a century it has been generally believed that AA progresses invariably from early inflammation to later gangrene and perforation, and that emergency appendectomy is always required for surgical source control [4].

Although non-operative management with antibiotics of uncomplicated acute diverticulitis and salpingitis has been well established, the non-operative management of AA remains controversial. There is one Cochrane analysis [5], five meta-analysis [6-10] and some reviews [11,12] of non-operative treatment of AA. Although a non-surgical approach in AA may reduce the complication rate, the lower efficacy may prevent antibiotic therapy from being a first-hand alternative to surgery [8]. On the other hand, appendectomy may not be always necessary for the patients with uncomplicated AA, as many patients resolve spontaneously and others may be treated with antibiotic therapy [13-17]. Six randomized controlled trials (RCTs) have compared the efficacy of antibiotic therapy with surgery in the treatment of AA [13-18].

Abdominal computed tomography (CT) is the best non-invasive diagnostic tool available and it has become more commonly used in this respect for patients with AA with a high sensitivity and specificity [19,20]. Most previous RCTs comparing antibiotic therapy with surgery in the management of AA are lacking abdominal CT to confirm AA [13-16]. Therefore, a well-designed controlled trial comparing non-operative management versus early appendectomy for uncomplicated AA corroborated by CT imaging has been called for [8]. CT scan is used in the APPAC trial for research purposes as CT scan confirmed uncomplicated acute appendicitis will prevent bias in our result as the antibiotic group patients are also treated for acute appendicitis enabling accurate comparison with the surgery group. The only previous study of antibiotic treatment in CT scan diagnosed AA indicated that amoxicillin/clavulanic acid was not non-inferior to emergency appendectomy in the treatment of AA, but identification of predictive markers, such as appendicolith, on CT scans might enable improved targeting of antibiotic treatment [17]. CT scanning of patients with suspected AA has been considered essential to exclude non-appendicitis and to identify perforated appendicitis or an appendiceal abscess reducing the number of non-therapeutic appendectomies and overall admission costs [19-22]. Meta-analysis and review articles suggest that although antibiotics may be used as the primary treatment for selected patients with suspected uncomplicated AA, this is unlikely to supersede appendectomy at present [6-10]. The recent meta-analysis by Mason et al. [8] identified non-operative management of uncomplicated AA to be associated with significantly fewer complications, better pain control and shorter sick leave, but overall having inferior efficacy because of high rate of recurrence (10 – 20%) in comparison with appendectomy.

### Objective

The objective of the APPAC trial is to compare antibiotic therapy (ertapenem) with emergency appendectomy in the treatment of CT scan confirmed uncomplicated AA. The overall objective of the study is to provide level I evidence to support the hypothesis that approximately 75–85% of patients with uncomplicated AA can be treated without surgery by using effective antibiotic therapy.

The primary endpoint will be the success of the randomized treatment. In the antibiotic treatment arm successful treatment is defined as being discharged from the hospital without the need for surgical intervention and no recurrent appendicitis during a minimum follow-up of one-year (treatment efficacy). Treatment efficacy in the operative treatment arm is defined as successful appendectomy evaluated to be 100%. Secondary endpoints are post-intervention complications, overall morbidity and mortality, the length of hospital stay and sick leave, treatment costs and pain VAS-scores.

## Methods/Design

### Trial design

The APPAC trial has been designed as a prospective randomized controlled, open label, non-inferiority multicenter trial to compare antibiotic therapy (intravenous ertapenem) with emergency appendectomy in the treatment of uncomplicated appendicitis.

### Participants

Patients presenting with suspected uncomplicated AA will be enrolled from six participating Finnish hospitals; three university hospitals and three central hospitals. The university hospitals are Turku, Tampere and Oulu University Hospitals, and the central hospitals are Mikkeli, Jyväskylä and Seinäjoki Central Hospitals.

All adult patients (aged 18 – 60 years old) admitted to the emergency department with a clinical suspicion of uncomplicated AA will be studied carefully by attending surgeons at the emergency departments of the participating hospitals. Clinical history, physical investigation and laboratory blood tests (blood hemoglobin g/l and leukocyte count E9/l, plasma C - reactive protein mg/l and creatinine μmol/l and serum human chorionic gonadotropin U/l) as well as urine analysis are undertaken. Before any pain medications are administered at emergency ward, pain scores (VAS 0–10) will be recorded. If clinical history and physical examination suggest that the patient has uncomplicated AA, the patient is eligible for inclusion in the APPAC study and the patients are informed of the protocol and invited to participate. After signed informed consent is obtained, a CT scan will be performed to confirm the diagnosis of uncomplicated AA.

Inclusion criteria 

▪ Signed informed consent

▪Age between 18 and 60 years

▪ CT scan diagnosis of uncomplicated AA

Exclusion criteria 

▪ Age < 18 years or > 60 years

▪ Pregnancy or lactating

▪ Allergy to contrast media or iodine

▪ Renal insufficiency, serum creatinine > 150 μmol/l

▪ Metformine medication

▪ Peritonitis

▪ Inability to co-operate and give informed consent

▪ Serious systemic illness

▪ Complicated AA in a CT scan: Appendicolith, perforation, periappendicular abscess or suspicion of a tumour

### Registration procedure

After signed informed consent, all patients evaluated for study enrollment are registered in every participating institution using the same data collection sheet. The patient namecode, date of birth, sex, eligible criteria and names of responsible physicians will be registered along with the clinical information. The data collection sheets will be combined into a common database at the main research center Turku University Hospital.

### Randomization

After confirming the diagnosis of uncomplicated AA by a CT scan, patients will be randomized by a closed envelope method either to undergo appendectomy or to receive antibiotic therapy with intravenous ertapenem. The randomization is performed in 1:1 equal allocation ratio. The 610 opaque, sealed, and sequentially numbered randomization envelopes are mixed and distributed to research hospitals by the main research center according to each hospital district population. To randomize a patient, an independent surgeon on duty will open the next consecutively numbered envelope.

### Interventions

#### Surgical treatment

After randomization to undergo operative treatment, open appendectomy will be performed by standard technique using a McBurney right lower quadrant muscle splitting incision. Prophylactic antibiotic as a single dose of 1.5 g cefuroxime and 500 mg metronidazole is administered approximately 30 min preoperatively. The histopathological examination of the appendix will be performed and the histological diagnosis of acute appendicitis requires involvement of the muscularis of the appendix (transmural neutophil invasion).

#### Antibiotic therapy

After randomization to receive antibiotic treatment, intravenous ertapenem sodium 1 g per day will be administered for three days with the first dose given in the emergency room. The clinical status of the antibiotic group patients will be re-evaluated within 12 – 24 hours after admission and monitored during the whole stay. If progressive infection, perforated appendicitis or peritonitis is clinically suspected, the patient will undergo emergency appendectomy and the histopathological examination of the appendix will be performed. In case of ertapenem allergy (known or newly diagnosed), the intravenous antibiotic treatment will consist of tazobactam 4 g × 3 combined with metronidazole 500 mg × 3. The three-day intravenous antibiotic treatment will be followed by seven days of oral antibiotic therapy with levofloxacin 500 mg × 1 combined with metronidatzole 500 mg × 3 resulting in ten-day total duration of the antibiotic therapy. In case of allergy for fluoroquinolones (known or newly diagnosed), levofloxacin will be replaced either with cefalexin 500 mg × 3 or clindamycin 400 mg × 3.

### Outcome parameters

#### The primary end-point

Success of the randomized treatment

The primary endpoint of treatment success in this non-inferiority trial is defined in the antibiotic treatment arm as the resolution of AA with antibiotic treatment resulting in discharge from the hospital without the need for surgical intervention and no recurrent appendicitis during a minimum follow-up of one-year (treatment efficacy). Treatment efficacy in the operative treatment arm is defined as successful appendectomy evaluated to be 100%.

Secondary end-points 

▪ Post-intervention complications

▪ Late recurrence of AA after conservative treatment

▪ Duration of hospital stay

▪ Treatment costs

▪ Post-intervention pain scores (VAS 0–10) and use of pain medication

▪ Sick leave

A recurrent AA will be diagnosed on a clinical basis. A patient with recurrent AA will always undergo appendectomy and the recurrent AA diagnosis will be verified by surgery and histopathological examination of removed appendix. For the primary study endpoint, the overall treatment efficacy will favor surgical treatment. For the secondary end-points, late recurrence of AA after one-year follow-up is naturally associated only with the antibiotic treatment arm. The outcome regarding the other secondary endpoints of overall morbidity, sick leave, treatment costs, pain scores and pain medication utilization in the antibiotic treatment arm is evaluated to be superior compared with surgical treatment. The duration of the hospital stay will most likely be similar in both treatment arms as the hospitalization of antibiotic group patients is protocol-driven in the trial design to ensure the safety of this unproved therapeutic modality.

#### Pre-intervention data

▪ Date of birth

▪ Sex

▪ Surgeon on duty

▪ Pain score (VAS) on admission

▪ Hemoglobin

▪ Leukocyte count

▪ CRP (C-reactive protein)

▪ Creatinine

▪ Human chorionic gonadotropin

▪ Urine analysis

▪ CT-scan data (see abdominal computed tomography)

▪ Informed consent and patient information

▪ Randomization

#### Intervention data

Surgical treatment 

▪ Antibiotic prophylaxis

▪ The timing of the operation and reasons for possible operative delay

▪ Operative findings

▪ Possible peroperative perforation of the appendix

▪ Operating time

Antibiotic therapy 

▪ The administered intravenous antibiotic

▪ Clinical status within 12 – 24 hours after admission and the surgeon performing the evaluation

▪ Possible cross-over to operative treatment and the clinical symptoms necessitating emergency appendectomy

▪ Adverse reactions to antibiotics

#### Post-intervention data

Surgical treatment 

▪ Clinical wound infection (surgical site infection, SSI) occurring within 30 days after the operative procedure diagnosed by a surgeon or positive bacterial culture 

○Superficial incisional SSI – infection involves only skin and subcutaneous tissue of incision presenting with at least one of the following signs or symptoms of infection 

▪ purulent drainage from the superficial incision

▪ pain or tenderness

▪ localized swelling

▪ redness or heat

○Deep incisional SSI – infection involves deep tissues, such as fascial and muscle layers 

▪ purulent drainage from the deep incision

▪ deep incision is deliberately opened by a surgeon in case of fever and localized pain or the incision spontaneously dehisces

○Organ/space SSI – infection involves any part of the anatomy in organs and spaces other than the incision, which was opened or manipulated during the operation

▪ Postoperative antibiotic treatment (at the hospital and after discharge)

▪ Pain score (VAS) on discharge date

▪ Profession

▪ Sick leave

▪ Pain medication prescription

#### Follow-up

Patient outcome will be obtained during hospital stay (days 0, 1, 2) and then by a phone interview at one week, two months and at and one, three, five and ten years after the intervention. At one week and two months pain score (VAS), possible additional need for sick leave, wound infections and recurrent AA will be registered. At long-term follow-up of 1, 3, 5 and 10 years recurrent AA and possible occurrence of appendiceal or cecal tumors will be registered for the antibiotic therapy arm and possible incisional hernias or other problems with the McBurney incision for the surgery group. Potential adhesion related problems will be evaluated for both study groups.

### Abdominal computed tomography

All abdominal CT scans will be performed from the diaphragm to the pubic symphysis using multi-detector row helical CT scanners (MDCT). A study series with contrast is performed during portovenous phase according to standard imaging protocol. The radiation dose of CT is set to be 6.7 mSv (range 5–7 mSv) depending on the size of patient.

Normal appendix is 6 mm or less in diameter in < 60% of patients [23]. The CT diagnosis of AA is based on the diameter of the appendix exceeding 6 mm, thickening and contrast enhancement of the appendiceal wall, inflammatory edema and minor fluid collection around the appendix. A standardized radiology data sheet is recorded for all patients undergoing a CT scan for a suspected AA evaluated for participation in the trial. A final CT diagnosis of uncomplicated AA requires a clear visualization of the appendix presenting with the previously stated radiological criteria of AA and the absence of any of following CT scan findings resulting in the diagnosis of complicated AA: 

▪ Periappendiceal abscess

▪ Perforated AA (periappendiceal abscess, extraluminal gas, free peritoneal fluid, focal poor enchancement of the appendiceal wall)

▪ The presence of appendicolith

▪ Tumour of the appendix

### Sample size calculation

The sample size calculation of the trial was based on the self-evident fact that the efficacy of appendectomy as a treatment for AA is 100%, but antibiotic therapy will not provide adequate source control in all patients with uncomplicated AA. However, the hypothesis of the APPAC trial is that operative treatment of uncomplicated AA is not mandatory for the majority of patients as 75 – 85% of patients with uncomplicated AA can be cured with wide-spectrum antibiotics avoiding a large number of unnecessary appendectomies [8]. For the primary endpoint of treatment success for the randomized therapy tested in a randomized, controlled, open label, non-inferiority multicenter trial, we assumed 99% healing rate of AA in the appendectomy group vs. 80% success rate for the antibiotic therapy. A non-inferiority margin of 24 percentage points was used in the sample size calculations meaning that the lower limit of the success in antibiotic therapy would be 75%. We calculated that a sample size of 275 patients per group would give a power of 0.9 (1-β) to establish whether antibiotic treatment was not inferior to appendectomy evaluated by treatment success in both study arms (significance level of 0.05 α). With an estimated 10 percent of the trial patients lost to follow-up, a maximum of 610 patients will be enrolled. For the secondary endpoints data will be compared as superiority trial setting and in superiority tests a two-tailed P value ≤ 0.05 will be considered statistically significant. The main analyses will be based on the intention-to-treat principle, but both intention-to-treat and per-protocol analyses will be performed.

### Cost analysis

All related costs will be estimated based on the actual input terms of resource use and personnel in the 12-month follow-up period after randomization. All costs will be derived from the Finnish hospital cost or determined in co-operation with the hospital administration. Direct medical costs will be recorded in the case record forms. Indirect costs arising from losses in productivity will be assessed by means of the Health and Labor questionnaire and will be calculated by means of the friction cost method.

### Safety monitoring

Adverse events are defined as any undesirable experience occurring to a subject during a clinical trial, whether or not considered related to the investigational intervention. All adverse effects reported spontaneously by the subject or observed by the investigator or the staff will be recorded. An interim analysis to ensure safety of the antibiotic treatment will be performed after randomizing 150 – 200 patients.

The radiation exposure caused by abdominal CT is 6 – 8 mSv. One mSv corresponds to four months background radiation exposure. An abdominal CT scan, with an estimated effective maximum dose of 10 mSv, raises the possibility of x-ray induced fatal cancer by 0.05%, in addition to a base-line life time risk for naturally induced fatal cancer of 20% in the U.S. [24]

### Ethics and informed consent

This study will be conducted in accordance with the principles of the Declaration of Helsinki and ‘good clinical practice’ guidelines. The Medical Ethical Committee of the Turku University Hospital has approved the protocol and the Ethical Committees of the participating centers are applied for local feasibility. Prior to CT scan evaluation and randomization, written informed consent will be obtained from all patients.

## Discussion

The hypothesis of the APPAC trial is that the majority of patients with uncomplicated AA can be cured with wide-spectrum antibiotics avoiding a large number of unnecessary appendectomies and this hypothesis is supported by previous randomized studies [13-18]. Acute appendicitis is one of the most common urgent conditions seen in general surgery practice. Although the exact mechanisms leading to this condition are still obscure, it is likely that luminal obstruction by external (lymphoid hyperplasia) or internal (sticked fecal material, appendicolith) compression plays a key pathogenetic role. The luminal obstruction leads to increased mucus production, bacterial overgrowth, and stasis, which increase appendiceal wall tension. Consequently, blood and lymph flow is diminished, and necrosis and perforation follow. As these events occur over time, it is conceivable that early surgical intervention prevents progression of disease. However, epidemiologic studies on incidence of nonperforated and perforated AA suggest that nonperforated and perforated AA may have different pathogenetic mechanisms strongly supporting our study hypothesis in re-evaluating the dictum that surgical removal of the appendix is always necessary for AA [25].

The best design for a therapeutic trial is a randomized placebo-controlled, double-blind study, but with the interventions used in the APPAC trial the concealment would not be possible and therefore a randomized open design was chosen. As concealment is lacking in all randomized trials comparing appendectomy with antibiotic therapy, the main focus should be on the safety of antibiotic treatment and the reduction in surgically-related morbidity and cost savings by using antibiotic therapy. Our power analysis and study hypothesis are based on the self-evident fact that efficacy of surgical treatment will be clinically superior to antibiotic therapy for uncomplicated AA – no appendix, no appendicitis – and therefore the primary end-point is treatment efficacy in both study arms. The primary endpoint of 30-day post-intervention peritonitis in the study of Vons et al. [17] is not clearly defined and, in addition, the definition varies between treatment arms. In the study by Hansson et al. [14] nearly half of the patients randomized to antibiotic group crossed over to the appendectomy group prior to receiving any drug and were classified as antibiotic treatment failures. Regarding these study designs, particular attention should be made to identify a clear and concise definition of efficacy to be used for both the conservative and surgical treatments, standardizing the different treatment procedures as much as possible [6-8] even though there is an intrinsic difficulty in defining a common outcome for both treatment arms.

Before enrolling patients into a randomized trial, the diagnosis of AA needs to be confirmed by CT, but this inclusion criterion has been used so far in only one study [17]. In contrast to this study by Vons et al. [17], we have determined the presence of intraluminal appendicolith as an important exclusion criterion, as it has earlier been reported to predict negative outcome of non-operative management and to predict complicated AA [26]. Indeed, if Vons et al. had excluded the patients with an appendicolith from their analysis, no significant difference in the incidence of post-intervention peritonitis between the treatment groups would have been noticed in their study.

The antibiotic therapy has been suboptimal in many previous randomized studies, as for example in the study by Vons et al. [17] amoxicillin-clavulanic acid was used even though this combination has been associated with considerable *Escherichia coli* non-susceptibility. Furthermore, the use of this combination may play a role in both the initial antibiotic treatment failures and the recurrence of AA considering that this antibiotic treatment is not recommended to be used in the non-operative treatment of AA [8,22]. The most common organism in AA is *Escherichia coli*, and the next most common is *Enterococcus* and other *Streptococcus* species. *Pseudomonas*, *Klebsiella*, and *Bacteroides* species are less commonly isolated. Accordingly, the selection of antibiotics should cover both aerobic and anaerobic bacteria [8,22,27]. In the present study ertapenem was chosen for the antibiotic therapy, because it is is a broad-spectrum antibiotic with a single-dose daily administration and the efficacy of ertapenem monotherapy in serious intra-abdominal infections has been demonstrated [22].

The results of our interim analysis (n = 161) corresponded both with the hypothesis of our study and the sample size calculation. Vons et al. [17] reported a recurrence rate of 26% in the antibiotic group. However, 68% of the patients in their study did not require appendectomy supporting our study hypothesis, that the majority of patients (> 70%) with uncomplicated AA can be treated successfully with antibiotics and unnecessary appendectomies can be avoided resulting in reduced morbidity and mortality of surgical treatment of AA, enormous cost savings and allocation of surgical resources to other emergency operations. Since so far only a small number of RCTs (< 1000 patients) with somewhat impaired methodological quality are available, more well-designed RCTs are urgently needed to both conclusively define the role of antibiotic therapy in the management of uncomplicated AA and to assess the predictive markers for successful non-operative treatment of uncomplicated AA.

## Conclusion

The APPAC trial is a randomized controlled open-label multicenter study comparing emergency appendectomy with antibiotic therapy (intravenous ertapenem) in the treatment of uncomplicated acute appendicitis.

## Competing interest

The authors declare that they have no competing interests.

## Authors’ contributions

HP/PS and JG drafted the manuscript, PS, HP, JG, SH, KD, TR, PN, TR, MA, J-PM, JS and AJ participated in the design of the study, principal investigator PS, SH, and HP performed the sample size calculations. All authors edited the manuscript, read and approved the final manuscript.

## Pre-publication history

The pre-publication history for this paper can be accessed here:

http://www.biomedcentral.com/1471-2482/13/3/prepub
